# Angular ectopic pregnancy presenting as rupture of lateral wall of the uterus

**DOI:** 10.4103/0974-1208.38970

**Published:** 2008

**Authors:** Baldawa PS, Chaudhari HK

**Affiliations:** Department of Obstetrics and Gynaecology, Seth Gordhandas Sunderdas Medical College, King Edward Memorial Hospital, Parel, Mumbai, India

**Keywords:** Angular, ectopic, pregnancy

## Abstract

This is a case report of a 32-year-old woman, being treated for secondary infertility, with history of previous ectopic pregnancy, who presented to the emergency obstetrical room in a state of hypovolemic shock. A diagnosis of ruptured ectopic pregnancy was confirmed in view of history of 14 weeks amenorrhea with a positive urine pregnancy test and positive colpopunture. She was immediately shifted for an emergency exploratory laparotomy. Intraoperatively, the authors were surprised to encounter a right lateral wall rupture uterus and ~14 weeks foetus with the placenta lying freely in the peritoneal cavity. That was suggestive of a right interstitial ectopic which had grown up to 14 weeks, invaded the uterine cavity thus forming an angular ectopic, which ended up as the catastrophic event. The authors here wish to highlight that angular pregnancy is rare but it has catastrophic consequences including maternal mortality. Had the patient presented early, in view of history of previous ectopic, an ultrasonography and color Doppler would have been useful in early detection. And a fertility conserving management in the form of Methotrexate therapy or Selective Uterine artery embolization could have been done.

An interstitial ectopic pregnancy develops in the uterine portion of the fallopian tube. Angular pregnancy originates in the interstitial portion of the fallopian tube and then grows into the adjacent uterine cavity. Thus an angular pregnancy is a continuation of an interstitial pregnancy. While interstitial pregnancies account for only 2-4% of all ectopic gestations,[1–3] they cause a disproportionately high incidence of hemoperitoneum and shock, and the mortality rate is approximately twice that of other types of ectopic pregnancies.[4] There are reported cases of rupture at all gestational ages, including full term pregnancy with surgical delivery.[4]

## CASE HISTORY

**Figure 1:**
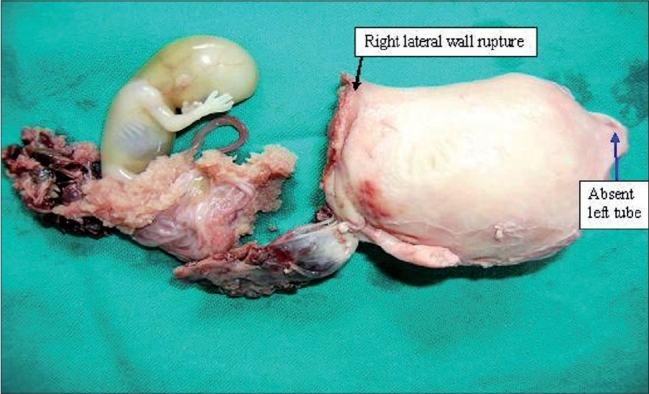
A right lateral wall uterine rupture and ~ 14 weeks foetus with the placenta

A 32-year-old woman, Gravida_3_ Parity_1_ Living_1_ ectopic_1_, presented to the emergency obstetrical room in a state of hypovolemic shock. Her pulse rate was 140/min BP of 70 mm Hg, RR of 30/min. There was history of 14 weeks amenorrhea, urine pregnancy test was positive, colpopunture revealed fresh red blood which did not clot, suggestive of hemoperitoneum. Relatives gave history of previous ectopic pregnancy for which left-sided salpingectomy was done 3 years back. Two large bore intravenous accesses were made and compatible whole blood transfusion and fluid resuscitation was started. She was immediately shifted for an emergency exploratory laparotomy. Intraoperatively, the authors encountered a right lateral wall rupture of the uterus and ~14 weeks foetus with the placenta lying freely in the peritoneal cavity, suggestive of rupture of a left angular ectopic pregnancy [Figure 1].

**Figure 2:**
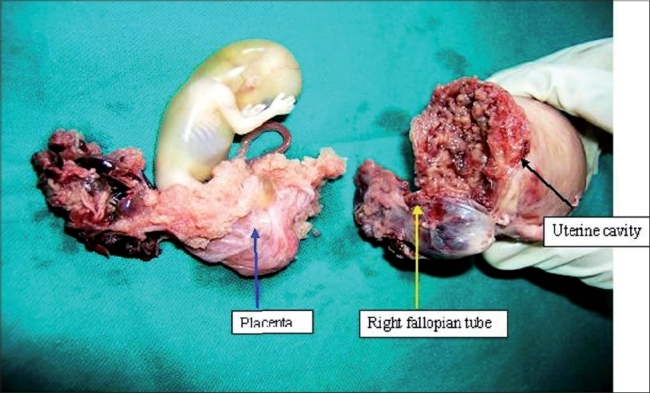
Subtotal hysterectomy with right salpingectomy

As the uterus could not be salvaged, subtotal hysterectomy with right salpingectomy was done [Figure 2] (left tube being previously removed).

She was shifted to the intensive care unit postoperatively. She required correction of her electrolyte imbalance and further blood transfusions during her recovery period.

She was discharged on 12^th^ postoperative day after complete recovery.

The authors here wish to highlight that angular pregnancy is rare, but it has catastrophic consequences including maternal mortality. Had the patient presented early and in view of her history of previous ectopic, an ultrasonography and color Doppler would have been useful in early detection and a fertility preserving conservative line of management in the form of Methotrexate therapy or Selective Uterine artery embolization could have been done.

## DISCUSSION

**Figure 3:**
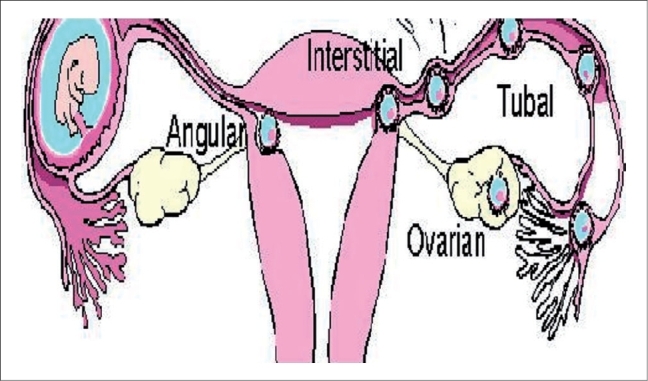
Classification of tubal ectopic pregnancies

An interstitial pregnancy is an uncommon type of ectopic pregnancy, accounting for 2-4% of all ectopic pregnancies.[1–3] The interstitial portion of the fallopian tube is a highly vascular, muscular site that offers more support and distensibility to the embryo than any other portion of the fallopian tube. These anatomic features allow the gestation to advance much further into its development than when the embryo implants in other portions of the tube [Figure 3]. A gestational sac surrounded by an incomplete or asymmetric uterine myometrial mantle is highly indicative of an interstitial pregnancy.[4,5] Color Doppler sonography reveals flow associated with the gestational sac. A spectral tracing reveals a high velocity-low resistance pattern, characteristic of trophoblastic blood flow.[6] The exact classification of the various types of ectopic gestations is somewhat controversial. Gestations implanted within the ampullary or isthmic portions of the fallopian tube are referred to as tubal ectopics.

Interstitial pregnancies occur when the gestation implants in the interstitial portion of the fallopian tube. While some consider the terms interstitial and cornual pregnancies to be synonymous, others reserve the term cornual pregnancy for a gestation in one horn of a bicornuate or septate uterus.[1–4] A seldom used term, angular pregnancy, refers to a gestation in which the pregnancy extends beyond the interstitium into the adjacent uterine cavity.[1,6]

In the case presented in this report, the gestation originated in the interstitial portion of the fallopian tube and extended into the adjacent uterine cavity, therefore was classified as an angular pregnancy.

## References

[1] Bond AL, Grifo JA, Chervenak FA, Kramer EE, Harris MA (1989). Term interstitial pregnancy with uterine torsion: Sonographic, pathologic and clinical findings. Obstet Gynecol.

[2] Maliha WE, Gonella P, Degnan EJ (1991). Ruptured interstitial pregnancy presenting as an intrauterine pregnancy by ultrasound. Ann Emerg Med.

[3] Coady DJ, Synder JR, Golstein SR, Subramanyan BR (1985). Ultrasound diagnosis of interstitial pregnancy. N Y State J Med.

[4] Jafri SZ, Loginsky SJ, Bouffard JA, Selis JE (1987). Sonographic detection of interstitial pregnancy. J Clin Ultrasound.

[5] Taylor KJ, Ramos IM, Feyock AL, Snower DP, Carter D, Shapiro BS (1989). Ectopic pregnancy: Duplex Doppler demonstration. Radiology.

[6] Shapiro RS, Garten AJ, Bogursky E (1992). Foetus: Interstitial pregnancy: Colour Doppler demonstration. Radiology.

